# The osteogenesis of Ginsenoside Rb1 incorporated silk/micro-nano hydroxyapatite/sodium alginate composite scaffolds for calvarial defect

**DOI:** 10.1038/s41368-022-00157-5

**Published:** 2022-02-14

**Authors:** Yuqiong Wu, Jiahui Du, Qianju Wu, Ao Zheng, Lingyan Cao, Xinquan Jiang

**Affiliations:** 1 Department of Prosthodontics, Shanghai Ninth People’s Hospital, Shanghai Jiao Tong University School of Medicine; College of Stomatology, Shanghai Jiao Tong University; National Center for Stomatology; National Clinical Research Center for Oral Diseases; Shanghai Key Laboratory of Stomatology; Shanghai Engineering Research Center of Advanced Dental Technology and Materials, Shanghai, China; 2 Stomatological Hospital of Xiamen Medical College, Xiamen, China

**Keywords:** Drug delivery, Stem-cell research

## Abstract

Ginsenoside Rb1, the effective constituent of ginseng, has been demonstrated to play favorable roles in improving the immunity system. However, there is little study on the osteogenesis and angiogenesis effect of Ginsenoside Rb1. Moreover, how to establish a delivery system of Ginsenoside Rb1 and its repairment ability in bone defect remains elusive. In this study, the role of Ginsenoside Rb1 in cell viability, proliferation, apoptosis, osteogenic genes expression, ALP activity of rat BMSCs were evaluated firstly. Then, micro-nano HAp granules combined with silk were prepared to establish a delivery system of Ginsenoside Rb1, and the osteogenic and angiogenic effect of Ginsenoside Rb1 loaded on micro-nano HAp/silk in rat calvarial defect models were assessed by sequential fluorescence labeling, and histology analysis, respectively. It revealed that Ginsenoside Rb1 could maintain cell viability, significantly increased ALP activity, osteogenic and angiogenic genes expression. Meanwhile, micro-nano HAp granules combined with silk were fabricated smoothly and were a delivery carrier for Ginsenoside Rb1. Significantly, Ginsenoside Rb1 loaded on micro-nano HAp/silk could facilitate osteogenesis and angiogenesis. All the outcomes hint that Ginsenoside Rb1 could reinforce the osteogenesis differentiation and angiogenesis factor’s expression of BMSCs. Moreover, micro-nano HAp combined with silk could act as a carrier for Ginsenoside Rb1 to repair bone defect.

## Introduction

Bone defect caused by injuries, tumor resection or osteoporosis resulted in severe dysfunction and interfered with people’s work and life remarkably. Bone mesenchymal stem cells (BMSCs), precursors of osteogenic cells, exhibit the potential capability of differentiating into osteoblastic cells and could be employed for the therapy of bone defect.^1^ To improve the efficiency of osteoanagenesis in patients, it’s crucial to stimulate the differentiation of BMSCs into osteoblasts. Recently, some traditional Chinese medicine have demonstrated their osteoinductive ability for BMSCs and dedicated the therapeutic properties for bone defects.^2^

Ginseng, as one of the herbal medicine, is widely used in a range of therapeutic and healthcare applications in China and other Asian countries.^3^ Ginsenoside is dammarane-type triterpene saponins acquired from ginseng and display a variety of pharmacology features like anti-cancer, anti-inflammatory, anti-oxidative and anti-apoptosis effect.^4–9^ These different pharmacology features are predominantly due to the steroid structure, which allows them to engage in interaction with cellular membranes, membrane-bound ionic channels, and exocellular and endocellular acceptors to generate changes at the transcriptomic level.^10^ Ginsenoside Rb1, the affluent ginseng saponin exists in ginseng roots, confers the pharmacology features, particularly in the heart and vessel system, endocrine system, and immunosystem.^10^ Some researches had reported that Ginsenoside Rb1 could inhibit the programmed cell death in isoproterenol-triggered cardiomyocytes, as well as the doxorubicin-triggered H9C2 cells.^11,12^ However, there was little study highlight the roles of Ginsenoside Rb1 in osteoblastic differentiation of BMSCs and bone defect repairing.

Herein, our team detected the osteoblastic differentiation of BMSCs induced by Ginsenoside Rb1. Furthermore, a local persistent releasing system of Ginsenoside Rb1 in the bone defect area was proposed. Hydroxyapatite (Ca_10_(PO4)_6_(OH)_2_, HAp) biological ceramic, as a naturally formed constituent of bony tissues, was well known to be bioactive and biocompatible in organisms, without antigenic characteristics and cytotoxicity.^11,12^ Our research has displayed that because of the outstanding specific surface area, micro-nano hybrid structured HAp (micro-nano HAp) particulates could be utilized as the carrier of drug delivery system to reinforce osteoinduction ability.^2^ Silk fibroin is a representative naturally formed biological polymer stemmed from Bombyx mori cocoons, and it possesses high versatility and minimal inflammatory reaction on account of its good biocompatibility and bio-degradability.^13^ However, the pure silk hydrogel was lack of osteogenic activity in vivo.^14^ In a previous study, with the addition of sodium alginate (SA), a polyanion co-polymer stemmed from brown sea algae, silk and Ca^2+^ could together generate an uniformed interpenetration aquagel to acquire a steady scaffold.^15^ Currently, the present research on using Ca^2+^ HAp, encapsulated with Ginsenoside Rb1, to cross-link both silk and SA to acquire steady dual net scaffolds utilized in bone defect was carried out to explore its osteogenesis effect. Based on the findings above, we aim to explore the therapeutic potential of Ginsenoside Rb1 as bone anabolic agents, as well as to establish a drug delivery system by taking advantage of micro-nano HAp and silk to load Ginsenoside Rb1, seeking to find a new guideline of biomaterials-drug-based healing strategies.

## Results

### Ginsenoside Rb1 maintained cell viability and inhibited apoptosis of BMSCs

**Fig. 1:**
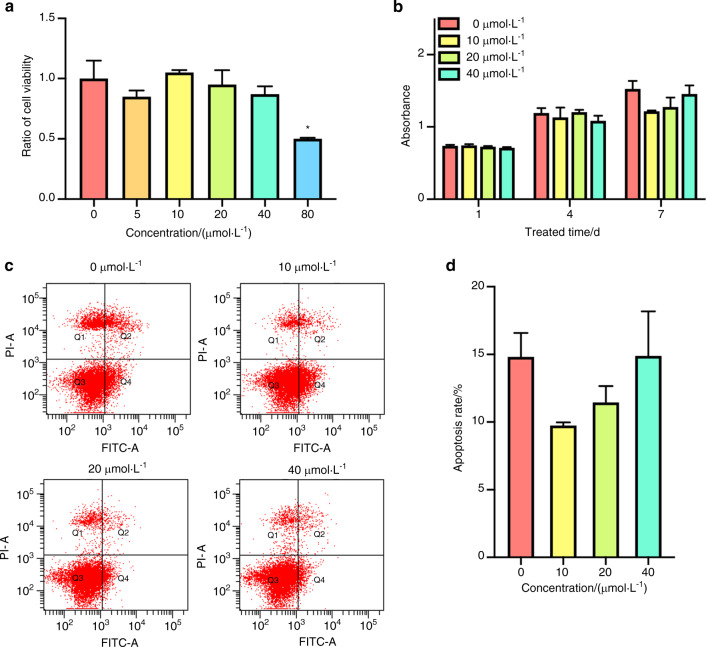
Proliferative ability and programmed cell death of BMSCs exposed to ginsenoside Rb1. **a** Cell toxicity assessment with diverse levels of ginsenoside Rb1. **b** Cell viability of BMSCs posterior to exposure to ginsenoside Rb1 by CCK8 analysis. **c**, **d** Apoptosis experiment of BMSCs. (*in contrast to 0 μmol·L^−1^ group at every temporal point, *P < 0.05; *n* = 3)

The cytotoxicity experiment was conducted to investigate the suitable level of Ginsenoside Rb1 for BMSCs and it turned out to be that the concentration of 80 μmol·L^−1^ were apparently excessive, leading to more than half BMSCs death (50.17% ± 0.47%) (*P* < 0.05) (Fig. 1a). Cell Counting Kit-8 (CCK8) assay showed that Ginsenoside Rb1 at 10–40 μmol·L^−1^ may have no superiority to enhance cell viability compared with 0 μmol·L^−1^ group (Fig. 1b). Meanwhile, Ginsenoside Rb1 exerted its advantages in reducing the apoptotic level in contrast to the controls. BMSCs apoptosis level treated with Ginsenoside Rb1 at 10 μmol·L^−1^ for 1 day were 9.74% ± 0.24%, while the control group was 13.83% ± 1.10% (*P* < 0.05, Fig. 1c, d), which exerted the advantage of Ginsenoside Rb1 on reducing the apoptotic level of BMSCs. Those outcomes revealed that the Ginsenoside Rb1 play an essential role in cell viability maintenance and apoptosis inhibition effect on BMSCs.

### Detection of Ginsenoside Rb1 in enhancing osteogenesis differentiation and angiogenesis factor expression of BMSCs

**Fig. 2:**
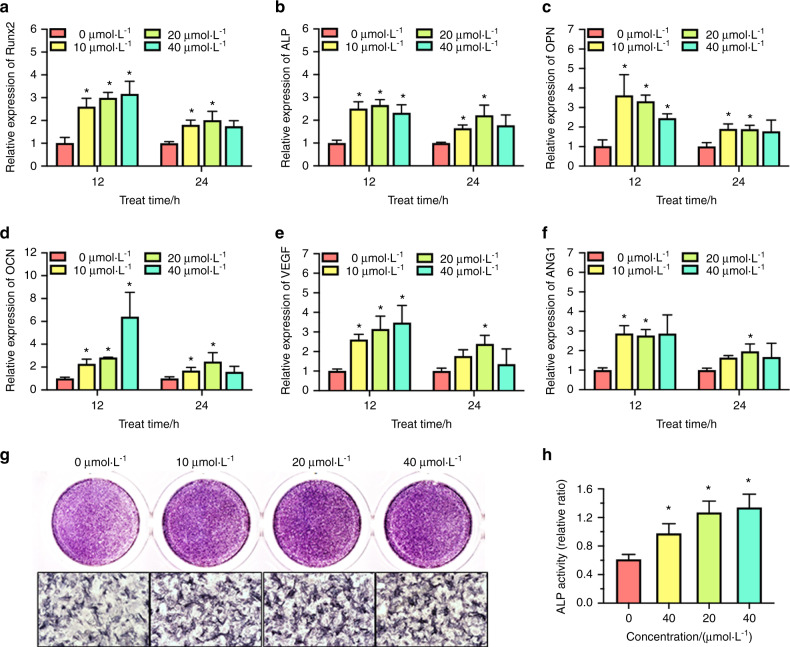
Gene expression of osteogenesis markers and ALP activity in BMSCs treated with ginsenoside Rb1. **a**–**f** Realtime PCR assay of Runx2, ALP, OPN, OCN, VEGF, and ANG 1 mRNAs in BMSCs exposed to ginsenoside (*in contrast to 0 μmol·L^−1^ at every temporal point). **g**, **h** ALP staining and ALP activity quantitative results after treatment with ginsenoside for 7 days (*in contrast to 0 μmol·L^−1^ group, *P < 0.05, *n* = 3)

In this study, the mRNA expressing of runt-related transcription factor 2 (Runx2), alkaline phosphatase (ALP), osteocalcin (OCN), vascular endothelial growth factor (VEGF), Angiopoietin-1 (ANG-1) in BMSCs was detected by the treatment of Ginsenoside Rb1 at the concentrations of 10, 20, and 40 μmol·L^−1^ (Fig. 2a–g). Ginsenoside Rb1 enhanced the mRNA expression of Runx2, ALP, OCN, and OPN of BMSCs after 12 h of treatment, and such elevating trend slowed down as the treating duration prolonged, but still higher than the control group, except the 40 μmol·L^−1^ groups (Fig. 2a–e). Similarly, the consistent expression pattern of the angiogenic factor VEGF and ANG-1 could be found (Fig. 2f, g).

Moreover, the ALP staining revealed that ALP activities were upregulated by Ginsenoside Rb1 at 7 days significantly (Fig. 2h). Meanwhile, ALP quantitative activity assay showed Ginsenoside Rb1 at 10, 20, and 40 μmol·L^−1^ concentration could raise the ALP activity in contrast to the controls at day 7 (Fig. 2h). Taken together, the results proved Ginsenoside Rb1 was of great potential in promoting osteogenesis differentiation, as well as angiogenesis factor expressing of BMSCs.

### ERK and AKT signal paths involved in the Ginsenoside Rb1-stimulated osteogenic differentiation

**Fig. 3:**
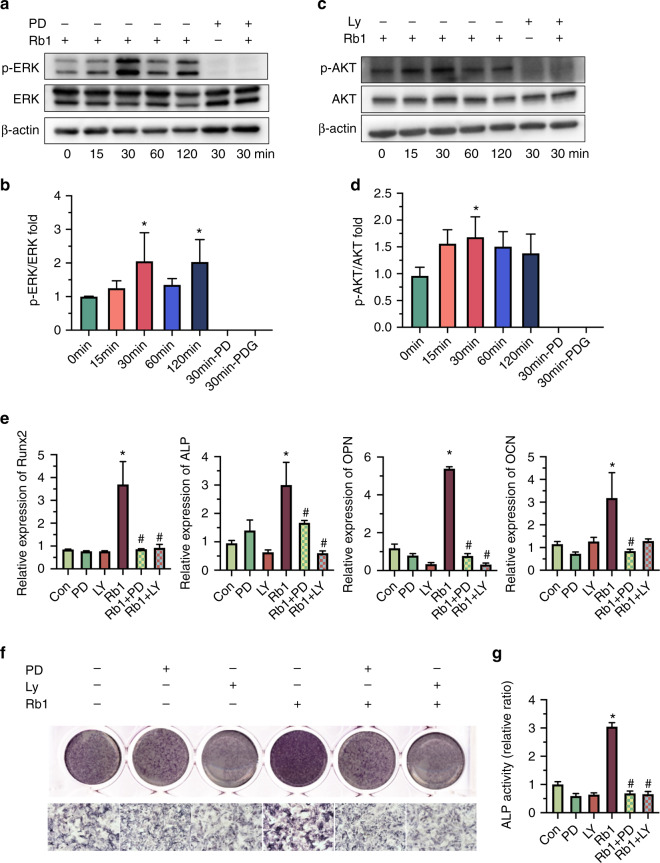
Roles of ERK and AKT signal paths in the osteogenesis differentiation of BMSCs induced by ginsenoside Rb1. **a**, **c** WB of phosphorylated and total ERK and AKT after ginsenoside Rb1 exposure at 0, 15, 30, 60, and 120 min and the treatment of ERK inhibitor PD98059 (20 μmol·L^−1^, PD in short) or AKT inhibitor LY294002 (20 μmol·L^−1^, LY in short). **b**, **d** Densitometry assay of p-ERK/ERK and p-AKT/AKT expression in western blot assay. **e** Realtime PCR assay of osteogenesis and angiogenesis gene expression of BMSCs posterior to the exposure to ginsenoside Rb1 with PD and LY. **f**, **g** ALP dyeing and quantitation analysis . (*^,^^#^*P* < 0.05; *in contrast to Con group, # in contrast to ginsenoside group)

To reveal the role of the ERK and AKT signal paths in the functioning of Ginsenoside Rb1, we investigated the total and phosphorylation levels of ERK and AKT under 20 μmol·L^−1^ concentration at the time of 0, 15, 30, 60, and 120 min. Results of western blotting showed that AKT and ERK were both phosphorylated posterior to Ginsenoside Rb1 (20 μmol·L^−1^) induction at the first 15 min, and peaked at 30 min. Then, they gradually fall back. As for ERK signaling, it had an extra high wave at the time point of 120 min (Fig. 3a, b). Meanwhile, it showed that p-ERK, as well as p-AKT triggered by Ginsenoside Rb1, were inhibited after the treatment of ERK signal path suppressor PD98059 or AKT signal path suppressor LY294002, separately, for 3 days (Fig. 3a–d).

To investigated the effects of ERK and AKT signal paths on Ginsenoside Rb1 activated bone regeneration, BMSCs were cultivated in the intermediary added with PD98059 or LY294002 separately for 7 days. The outcomes of realtime PCR demonstrated that the higher expression of osteogenesis genes induced by Ginsenoside Rb1 were significantly downregulated by PD98059 and LY294002, respectively (Fig. 3e). ALP dyeing and quantitation determination results revealed that the enhanced ALP activities in BMSCs triggered by Ginsenoside Rb1 was inhibited posterior to PD98059 or LY294002 treatment (Fig. 3f, g). The results strongly suggest that Ginsenoside Rb1 induced the osteogenesis differentiation of BMSCs, at least partially, through the stimulation of ERK and AKT signaling pathway.

### Ginsenoside Rb1 promotes the homing for HUVECs and BMSCs and promotes capillary tube forming of HUVECs

**Fig. 4:**
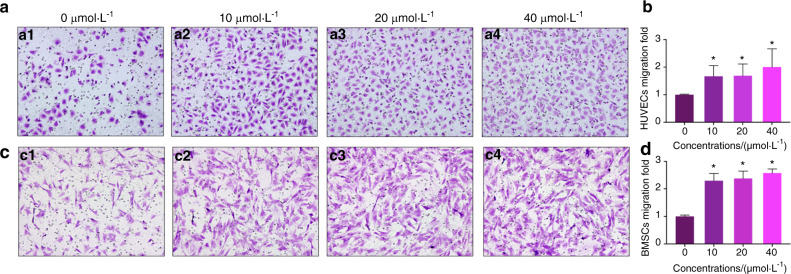
Transwell experiment was conducted to identify the migratory ability of HUVECs and BMSCs affected by Ginsenoside Rb1. **a**, **c** HUVECs and BMSCs were seeded on the upper chamber and the medium containing different concentrations of Ginsenoside Rb1 was placed in thelower chambers. After 24 h incubation, cells that traversed the membrane were stained with 0.1% crystal violet and imaged by a microscope (200×). **b**, **d** are quantitative analyses of **a** and **c**, respectively (*P < 0.05).

**Fig. 5:**
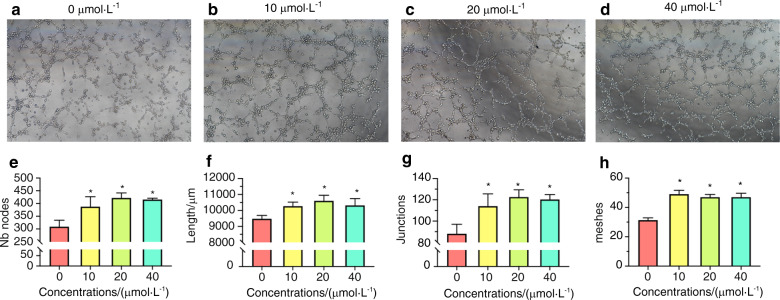
Angiogenesis test based on Matrigel was completed to identify the quantity of vessel-like tubes generated in vitro. **a**–**d** Microscopy images of HUVECs after incubation with different concentrations of Ginsenoside Rb1on Matrigel for 300 min at 37 °C (100×). **e** Nb nodes, **f** length, **g** junctions, and **h** meshes are quantified, respectively (*P < 0.05).

To explore the potential of Ginsenoside Rb1 in the recruitment of human umbilical vein endothelial cells (HUVECs in short) and BMSCs, the homing capability of Ginsenoside Rb1 for HUVECs and BMSCs was tested. The transwell migration test result showed that Ginsenoside Rb1 at the levels of 10, 20, and 40 μmol·L^−1^ could facilitate the motility of HUVECs and BMSCs significantly in vitro, as shown in Fig. 4. Since the capillary tube net forming is vital for angiogenetic activities, capillary tube formation ability of HUVECs with the treatment of Ginsenoside Rb1 at concentrations of 10, 20, and 40 μmol·L^−1^ were investigated and overall capillary tube length and the quantity of branching points per field were quantified as presented by Fig. 5. Vessels were generated within a capillary net under Ginsenoside Rb1 at concentrations of 10, 20, and 40 μmol·L^−1^ obviously. In contrast, HUVECs in control group without Ginsenoside Rb1 failed to generate vessels posterior to 5 h on growth factor-decreased matrigel. Notably, number of branch nodes, length, junctions, and meshes per field were increased, in contrast to the controls, demonstrating the underlying angiogenesis of Ginsenoside Rb1.

### Detection of micro-nano HAp granules

**Fig. 6:**
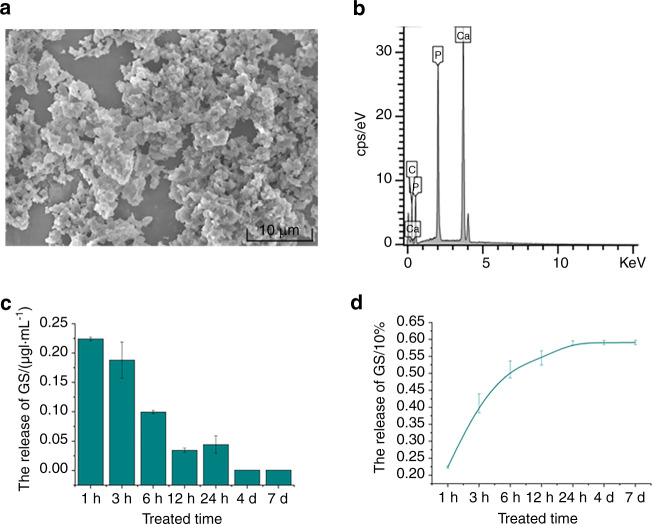
Features of micro-nano HAp particulates. **a** The SEM images of micro-nano HAp particulates (scale bar = 10 μmol·L^−1^). **b** The elements on the scaffold surface were analyzed. **c**, **d** The cumulative releasing proportion (%) of ginsenoside Rb1 loaded in the micro-nano HAp particulates at 2000 μmol·L^−1^

The acquired micro-nano HAp particulates displayed irregularity morphologically in shape under the micrographs with low magnification(Fig. 6a, b). The energy-dispersive X-ray spectrometry (EDS-XRS; QUANTAX 400-30, BRUKER, Karlsruhe, Germany) was adopted to analyze the elements on the scaffold surface. It showed that granules detected contained calcium (37.88% in wt), phosphorus (16.82%), carbon (19.98%), and oxygen (25.32%) (Fig. 6c), which was the same as the calcium to phosphorus ratio of hydroxyapatite.

The Ginsenoside Rb1 released from particulates was displayed in proportion (%). Figure 6d, e demonstrates that the accumulative releasing of Ginsenoside Rb1 at 2000 μmol·L^−1^ when cultivated in vitro. At the first 60 min, a burst releasing efficiency for micro-nano HAp/Ginsenoside Rb1 groups was identified, maintained at about 22.5%. After that, Ginsenoside Rb1 was produced in a linearity upward tendency at the first 6 h, and then released at a slow rate. After 24 h, Ginsenoside Rb1 was released slowly into the plateau, and reached 59.12% ± 0.63% at day 7 eventually. As per those outcomes, it revealed that micro-nano HAp, as a drug-delivering vehicle, could offer persistent release dynamics.

### Ginsenoside Rb1 promotes the TE, AL, and Ca deposition in fluorescent dye labeling histomorphometry assay

**Fig. 7:**
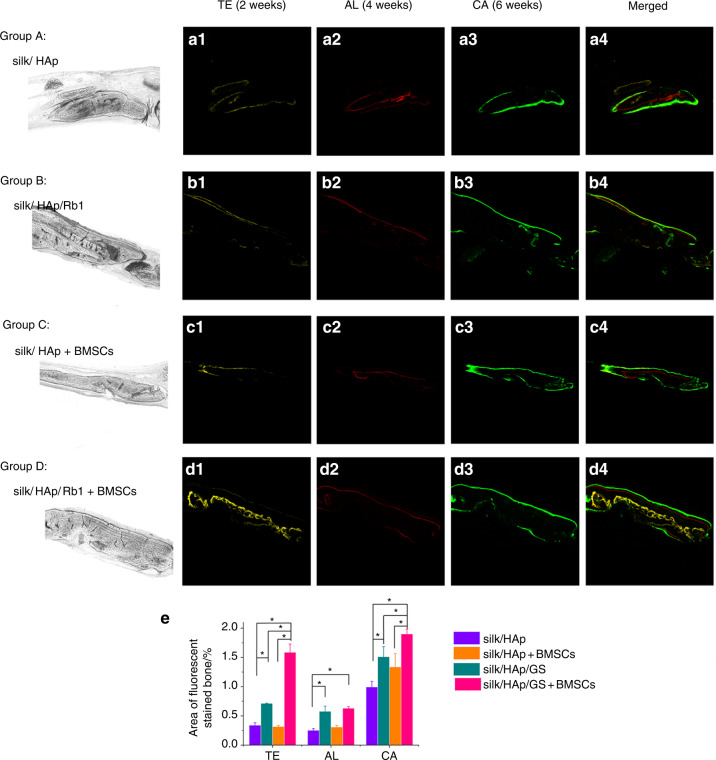
Sequence fluorescence labeling of TE, AL, and CA for groups silk/HAp, silk/HAp/Rb1, silk/HAp+BMSCs, and silk/HAp/Rb1+BMSCs. **a** The pictures in yellow (TE; **a1**, **b1**, **c1**, **d1**), red (AL; **a2**, **b2**, **c2**, **d2**) and green (CA; **a3**, **b3**, **c3**, **d3**) indicated the rate of calvariae forming and mineralisation at 2, 4, and 6 weeks posterior to operation, separately. **a4**, **b4**, **c4**, **d4** Meraged pictures of the 3 fluorescent dyes for the identical group. Scale bar = 100 μm. **e** The proportion (%) of TE, AL and CA dyeing via histomorphometry assay (*P < 0.05)

For the osteogenesis evaluation, fluorescence labeling assay was employed for the specimens to observe the bone regeneration in calvarial defects. As presented by Fig. 7, the diverse fluorescence labeling denoted osteogenesis and mineralisation at weeks 2, 4, and 6 after the operation, separately. At 2 and 6 weeks, the percentages of TE labeling (yellow), and Ca labeling (green) in the silk/HAp/Rb1+BMSCs group were highest (*P* < 0.05). At 2, 4, and 6 weeks, the percentages of TE labeling (yellow), AL labeling (red), and Ca labeling (green) in the silk/HAp/Rb1 group were greater in contrast to the silk/HAp group (*P* < 0.05). At 6 weeks, the percentage of AL labeling (red) in the silk/HAp+BMSCs group was greater in contrast to the silk/HAp group (*P* < 0.05).

### Ginsenoside Rb1 promotes bone regeneration in the histological analysis

**Fig. 8:**
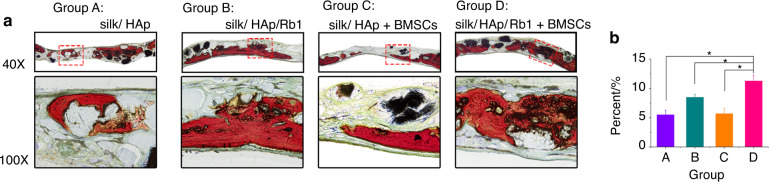
**a** Histology pictures of new bone formation in cranial defects for groups silk/HAp (Group A), silk/HAp/Rb1(Group B), silk/HAp+BMSCs(Group C), and silk/HAp/Rb1+BMSCs(Group D) were captured at the eighth week posterior to operation. **b** The proportion (%) of osteogenesis region evaluated at the eighth week posterior to implantation via histomorphometry assay (*P < 0.05; the first column: 40×; the second column: 100×)

Meanwhile, the non-decalcified samples dyed in van Gieson’s picro fuchsine (Fig. 8) displayed that the new bone formation was commencing after the operation. More new bone formation was identified within the groups of silk/HAp/Rb1 (8.52% ± 0.41%) and silk/HAp/Rb1+BMSCs (11.31% ± 0.97%), especially in the silk/HAp/Rb1+BMSCs group. Moreover, the new bone in the silk/HAp/Rb1+BMSCs group was more than the other three groups.

## Discussion

Traditional Chinese medicine has been inherited for thousands of years in China. As a reinforcing qi medicine, ginseng has been considered as the tonic of herbs. Ginsenosides, the active ingredients naturally present in ginseng, have a variety of beneficial effects on some disease, such as metabolic, vascular, and central nervous system disease.^10,16^ Particularly, Ginsenoside Rb1 contains a highly concentrated form of ginsenosides and exerts various pharmacological effects on metabolic disorders, including the modulation of its antioxidant, anti-inflammatory, anti-apoptosis effects, and promoting osteogenesis.^10,17^ Therefore, it was reasonable to speculate that Ginsenoside Rb1 might be a substitute for exogenous cytokines or growth factors to improve the therapeutic efficiency of bone defect.

Ginsenoside Rb1 has been shown to promote the proliferation of endogenous neural stem cells.^18^ This study found that Ginsenoside Rb1 at the concentrations of 10, 20, and 40 μmol·L^−1^ could maintain the viability of BMSCs, and reduced apoptosis cell ratio after 1 day’s treatment. However, some investigations demonstrated that Ginsenoside Rb1 inhibit cell activity of rat hepatic stellate cells,^19^ and this paradox attributed potentially to the inconsistent cellular response of different cell types and the different concentration of Ginsenoside Rb1. Moreover, it was notable to find out that Ginsenoside Rb1 had an excellent ability in improving osteogenesis differentiation of BMSCs. The osteogenesis function was demonstrated by the mRNA expressing of Runx2, ALP, OPN, OCN, and ALP activity. Runx2 showed an essential effect on modulating the expressing of osteoblastic genes at the early stage.^20^ The mRNA expression of Runx2 in 10, 20, and 40 μmol·L^−1^ Ginsenoside Rb1-treated groups was induced at 12 h and 24 h, except 40 μmol·L^−1^. OPN was related to the mature phase of osteoblastic cells in the period of attachment and substrate syntheses prior to mineralisation, and OCN was related to the matrix deposition and mineralisation. Their expressions were both enhanced after 12 h treatment, and fall back at 24 h, but still higher than the control group except 40 μmol·L^−1^. Some investigations demonstrated that the osteoblast differentiation has been promoted by Ginsenoside after 72 h of culture,^21^ while others reported that the osteoblasts differentiation have been enhanced by diverse level of Ginsenoside (10^−5^, 10^−6^, and 10^−7^ μmol·L^−1^).^22^ This phenomenon may be caused by the different experimental conditions and cell types tested in individual studies.

Although the data manifested the capability of Ginsenoside Rb1 in stimulating osteogenesis, we also wanted to determine the underlying mechanisms by which this phenomenon took up. The ERK signaling pathway had been demonstrated to modulate osteoblastic growth, programmed cell death and differentiative activities via modulating the expressing of cellular cycle regulators.^23^ Meanwhile, AKT signaling pathway was demonstrated to be pivotal for the physiological and pathophysiological process of many cellular types, and is vital for different activities, such as cellular growth, metabolic activity, motility, and differentiation.^24,25^ In the present study, ERK and PI3K/AKT were phosphorylated by Ginsenoside Rb1 rapidly. Furthermore, Ginsenoside Rb1-stimulated osteogenic gene expression and ALP activity of BMSCs were both weakened by either specificity ERK suppressor of PD98059 or AKT suppressor LY294002 dramatically. Other researchers also reported that hypoxia/reoxygenation-triggered programmed cell death in H9C2 cardiac muscle cells could be avoided by Ginsenoside by AKT, JNK, and ERK1/2 pathways,^26^ and the exposure to Ginsenoside on the AlCl_3_-triggered osteoblastic viability might rely on its role in the ERK, JNK, and AKT signal paths.^27^ These results were consistent with our research.

Biomaterials of HAp have been widely reported,^28^ and the scaffold of micro-nano HAp tend to show biocompatibility and repair ability in cranial defect of SD rats and could serve as a valuable candidate in the field of bone repair,^2^ laying a solid foundation for our present study. Silk fibroin (SF) has been accepted by U.S. Food and Drug Administration (FDA) in clinic application because of its attractive material properties such as superior biocompatibility, predictable degradability and non-inflammatory byproducts.^29,30^ And further, it could be fabricated into different material formats, including films, coating or spheres, providing a versatile biomaterial platform for the delivery of encapsulated drugs or growth factors.^31,32^ In the present study, we try to fabricate a delivery platform on the base of micro-nano HAp, together with SF incorporation, since no previous studies had systematically investigated the potential of such delivery system for sustained release profile, nor the application in vivo. According to previous study, silk and SA could generate a uniformed interpenetration aquagel simultaneously by the incorporation of calcium, which accelerated the forming of the β-sheet conformation of silk to trigger gelatinization.^18,33^ So it’s pivotal to identify a source of Ca that can be delivered within the system for the slow releasing of Ca, and avoid of aquagel nonuniformity induced by the overmuch releasing of Ca. Calcium silicate Hap (Ca^5^(PO_4_)^3^(OH)) was selected as the source of calcium, because of the satisfactory biocompatibility and degradation ability. Moreover, HAp granules could be micro-nano hybrid structured, and provided excellent specific surface area, which could be utilized as a carrier of drug release system to reinforce osteoinduction ability.^33,34^ Since the micro-nano HAp particulates, and the rod-like morphology of HAp particulates were capable of reticulating into a net, Ginsenoside Rb1 was capable of penetrating into the HAp particulates, which was capable of validly slowing down the releasing velocity. For that reason, it revealed that micro-nano HAp particulates exhibited a brilliant capability of controlling the Ginsenoside Rb1 delivery in vitro.

The early forming of novel vessel nets by HUVECs and the rapid bone regeneration by BMSCs of engineered constructs is pivotal for the successful result of bone defect restoration.^35^ Vessels produce a different metabolism and molecule micro environment, regulating the bone vasculature growth, sustaining perivascular osteoprogenitors, and they couple angiogenetic activities with osteogenesis.^36–38^ The homing experiment demonstrated that Ginsenoside Rb1 promoted the migration of HUVECs and BMSCs significantly. Moreover, a remarkable elevation in the quantity of tubule formation was found in the Ginsenoside Rb1-exposed groups, revealing that Ginsenoside Rb1 could promote the proliferation of HUEVCs, and facilitate subsequent angiogenesis.

Even although preliminary investigation indicated the potential ability of Ginsenoside Rb1 to promote osteogenesis and angiogenesis, the ultimate objective is to employ them in clinical application.^39^ Restoration of bone defect always needs filler ranging from autologous bone to heterogeneous bone, which including a series of shortcomings.^40,41^ In the present study, coinciding with the outcomes in vitro, Ginsenoside Rb1 encapsulation in the micro-nano HAp particulates elevated the capability of HAp in regulating the osteogenesis of the rat calvarial defect model. Moreover, with the addition of BMSCs, the treatment efficacy was significantly enhanced. Previous studies had well demonstrated that endothelial cells and mesenchymal stromal cells act as one of the key players in bone tissue regeneration and vascularization progress, and crosstalk between these cells attribute to the synergistic effects on regeneration.^36,42–44^ Hence, it is reasonable to assume that the migratory inducing ability Ginsenoside Rb1 increasing the number of HUVECs and BMSCs, which need a period of time to undergo biological progress at the fracture site and meanwhile, the addition of mature BMSCs could facilitate the rapid bone formation, which save time to some extent.

## Conclusion

In conclusion, the present study had provided more valuable evidence for traditional Chinese medicine. Ginsenoside Rb1 is of significant effectiveness in promoting cell proliferation and viability, inhibiting cell apoptosis and is conductive to osteogenesis. Ginsenoside Rb1 may be a candidate for exogenous cytokines or growth factors to improve the efficiency of therapy of bone defect patients. It may be a prospective approach for new bone formation.

## Methods and materials

### Cell culture and animal surgery

Four-week-old male Sprague-Dawley rats (Shanghai SLAC Experiment Animal Center, PRC), weighing about 70 g, were used for BMSCs isolation and culture as per the method described previously.^45^ In short, both ends of the Tibiae and femurs were severed and bonemarrow was acquired through flushing via DMEM (Hyclone, America) added with 100 unit per mL penicillin and 100 μg·mL^−1^ kyowamycin (Hyclone). Posterior to centrifugation under 1800 rpm for 600 s, the sediment was blended with full DMEM added with 10% FBS (Hyclone), and cultured at 37 °C in a humidified 5% carbon dioxide incubator. Nonadherent cells were discarded via the change of the new intermediary every 3 days. When big colonies were generated and became confluent, the primary rat BMSCs was passaged. The BMSCs from passage 2–3 were utilized for the assays.

HUVECs were chosen as the typical lineage cells herein to explore the bioactivity of GD on angiogenesis. HUVECs were grown in Endothelial Cell Medium (Sciencell, Carlsbad, CA, USA). The cultivation intermediary was added every 2–3 days and cells were cultivated when there was approximately 80% confluence.

For bone regeneration in situ experiments, 12 female Sprague-Dawley rats were conducted with critical-size cranial defect surgery. All animal experiments were accepted by the Ethical Board of our hospital affiliated to SJTU.

### Cytotoxicity evaluation (LD50)

BMSCs were inoculated onto 96-well dishes at 5 × 10^3^ cells per well. Posterior to a 24 h cultivation, they were exposed to 0, 5, 10, 20, 40, and 80 μmol·L^−1^ Ginsenoside Rb1, separately. At 24 h, the cell toxicity assessment was completed via MTT analysis. As per the supplier’s specification, 20 μL 5 mg·mL^−1^ MTT (Amresco, America) liquor was supplemented and cultivated under 37 °C for 240 min to generate MTT formazan. After that, the intermediary was substituted by 200 μL DMSO (Sigma, America) for the purpose of dissolving the formazan, and the absorption was identified at 590 nm via the ELX Ultra micro plate analyzer (Bio-tek, America). The proportion of viable cells was determined via contrasting the specimen absorption in the presence or absence of Ginsenoside Rb1.

### Cell viability and proliferation

The long-term effects of Ginsenoside Rb1 on the cellular viability and growth of BMSCs was estimated by Cell Counting Kit-8 (CCK-8) analysis. BMSCs were inoculated into 96-well dishes at 2 × 10^4^ cells per well. Posterior to a 24-h cultivation, they were exposed to Ginsenoside Rb1 at 10, 20, and 40 μmol·L^−1^ for 1, 4, and 7 days separately. The same volume of vehicle (1 μL DMSO/per mL intermediary) was supplemented to generate the controls (0 μmol·L^−1^). As per the supplier’s specification, cellular proliferative ability was evaluated via CCK-8 analysis. Every assay was completed at least for three times.

### Apoptotic experiment

An annexin V-FITC tool (Becton Dickinson) was employed to realize the quantification of programmed cell death. Posterior to the exposure to Ginsenoside Rb1 at the concentrations of 0, 10, 20, and 40 μmol·L^−1^, for 24 h respectively, the cells cultivated in 6-well dishes were collected, cleaned with PBS under 4 °C, afterward subjected to resuspension in 100 μL binding buffering solution with 5 μL annexin V-FITC and 5 μL PI. Posterior to a 15-min cultivation under room temperature, supplemented with other 400 μL binding buffer, stained cells were studied via flow cell technique. Every assay was completed at least for three times.

### Osteoblastic, angioblastic gene expression, and ERK, AKT inhibitor treatment analysis by qRT-PCR

BMSCs were placed onto 6-well dishes at 2 × 10^5^ cells per well, and cultivated for 24 h, before cultivation with Ginsenoside Rb1 at the final contents of 0, 10, 20, and 40 μmol·L^−1^, separately. Overall RNA was separated from the cells posterior to 12 h and 24 h Ginsenoside Rb1 exposure via the Trizol reagent (Invitrogen, America), as per the supplier’s specification. cDNA was prepared via a cDNA Preparation Reverse Transcriptional Tool (Fermentas, America). Realtime PCR analysis for Runx2, OPN, OCN, VEGF, and ANG-1 was completed via a Light-Cycler system through SYBR Pre-mix Ex TaqTM (Takara, Japan) as per the supplier’s specification. The parameters for realtime PCR were stated below: denaturating under 95 °C for 10 s; 50 cycles under 95 °C for 10 s and 60 °C for 30 s; and an eventual dissociating phase (95 °C for 300 s) supplemented at the end of the magnification process. β-Actin was utilized as the inner control. The data were studied via the comparative Ct (2−ΔΔCt) approach and were described as a fold change in contrast to the controls. Every assay was completed at least for three times. The primer sequences herein were presented by Table 1.

**Table 1:** List of primers used and respective forward and reverse sequences

Gene	Forward sequence	Reverse sequence
β-actin	5′-GTAAAGACCTCTATGCCAACA-3′	5′-GGACTCATCGTACTCCTGCT-3′
Runx2	5′-ATCCAGCCACCTTCACTTACACC-3′	5′-GGGACCATTGGGAACTGATAGG-3′
ALP	5′- TTTGCTACCTGCCTCACTTCCG-3′	5′-GGCTGTGACTATGGGACCCAG-3′
COL I	5′-CTGCCCAGAAGAATATGTATCACC-3′	5′-GAAGCAAAGTTTCCTCCAAGACC-3′
OCN	5′-GCCCTGACTGCATTCTGCCTCT-3′	5′-TCACCACCTTACTGCCCTCCTG-3′
OPN	5′-CCAAGCGTGGAAACACACAGCC-3′	5′-GGCTTTGGAACTCGCCTGACTG-3′
VEGF	5′-GGCTCTGAAACCATGAACTTTCT-3′	5′-GCAATAGCTGCGCTGGTAGAC-3′
ANG1	5′-GGACAGCAGGCAAACAGAGCAGC-3′	5′-CCACAGGCATCAAACCACCAACC-3′

To investigate the ERK and AKT signaling pathway, ERK and AKT inhibitor treatment analysis had been conducted. BMSCs exposed to Ginsenoside Rb1 at 0 and 20 μmol·L^−1^ were cultivated in the intermediary added with ERK signal path suppressor PD98059 (Beyotime), or AKT signal path suppressor LY294002 (Beyotime) for 7 days, at final concentration 20 μmol·L^−1^ and 20 μmol·L^−1^, separately. Overall RNA was separated and synthesized cDNA, and realtime PCR was completed on Runx2, ALP, OPN, and OCN as aforementioned.

### Alkaline phosphatase Staining and activity

BMSCs at 5 × 10^4^ cells per well were cultivated nightlong in 24-well dishes. ALP dyeing and activity quantitation were completed at the seventh day posterior to the exposure to Ginsenoside Rb1 at 0, 10, 20, and 40 μmol·L^−1^, separately. For the AKT and ERK suppressor exposure assay, BMSCs exposed to PD98059 (Beyotime) or LY294002 (Beyotime) for 7 days at final concentration 20 μmol·L^−1^ and 20 μmol·L^−1^, together with 20 μmol·L^−1^ Rb1. For ALP staining, all specimens were cleaned with PBS for 3 times and subjected to fixation with 4% paraformaldehyde for 600 s, and afterward cultivated in a matrix solution from an ALP dyeing tool (Beyotime), as per the supplier’s specification. After staining, the results were observed via a digital camera (ECLIPSETS 100, NIKON, Tokyo, Japan). For ALP activity quantitation, the cells were cleaned 3 times in PBS, and 200 μL of lysis buffering solution was supplemented into the cellular layer and maintained on ice for 20 min. The cellular lysate was subjected to sonication for 60 s and treated with centrifugation at 12,000 r·min^−1^ under 4 °C for 600 s. ALP activities were analyzed using Alkaline Phosphatase Assay Tool (Beyotime, Suzhou, PRC) as per the supplier’s specification. The OD at 405 nm were measured to determine ALP activities. Overall protein levels were evaluated via a Pierce^TM^ BCA Protein Analysis Tool (TFS, America). OD results were standardized to bovine serum albumin standard curve, at 562 nm. ALP activities were evaluated as OD results at 405 nm per mg of overall protein.

### ERK and AKT quantitation by western blotting

BMSCs were cultivated with the intermediary of 20 μmol·L^−1^ Ginsenoside Rb1 for 0, 15, 30, 60, and 120 min containing PD95059 or LY294002. At each time point, cells were subjected to lysis on ice for 0.5 h with RIPA lysis buffering solution (Beyotime) added with protease suppressor mix, phosphatase suppressor mix and phenylmethanesulphonyl fluoride (PMSF) (Beyotime, Suzhou, China). After the protein level was identified via a Pierce^TM^ BCA Protein Analysis Tool (Thermo Fisher Scientific), 20 μg was dissolved via 10% SDS-PAGE gel and electrotransferred onto polyvinylidene difluoride (PVDF) films (Pall, America). The films were subjected to blockade and cultivated with suitable first antisubstances, such as rabbit antirat ERK, AKT, p-ERK, and p-AKT (Cell Signaling Technology, Danvers, MA, America) at desaturation of 1:1000. For standardization of protein loading, mouse antirat β-actin (Abcam, Cambridge, MA, America) antisubstance was utilized at 1:10 000 desaturation. Eventually, the visualization of film reactions was realized via second antisubstances conjugated with horseradish peroxidase (Beyotime, desaturation, 1:1000) with ECL plus reagents (Amersham Pharmacia Biotech, UK) via UVItec ALLIANCE 4.7 gel image formation instrument. The density of ERK and AKT was subjected to quantification for protein expressing of p-ERK and p-AKT, respectively.

### Homing assay for HUVECs and BMSCs

In the homing assay for HUVECs and BMSCs, transwell migration tests (Corning Costar, America) were completed. In short, 3 × 10^5^ HUVECs or BMSCs were inoculated onto the upper insert with 8 μm apertures, and the lower chamber containing different concentrations of Ginsenoside Rb1. Posterior to a 24 h co-culture, the upper cells were removed, and the cells beneath the transwell were dyed with crystal violet and quantified analysis after dissolved by acetic acid.

### In vitro sprouting analysis

The in vitro sprouting assay was finished as described in the past.^28,29^ GF reduced Matrigel (BD Biosciences, America) was subjected to thaw under 4 °C and was added into 24-well plates on the ice. The dishes were afterward moved into an incubating device under 37 °C for 0.5 h to realize the gelation. Meanwhile, HUVECs were detached by trypsin and counted before resuspended in ECM with diverse levels of Ginsenoside Rb1. Then HUVECs were placed onto the gel at 10^5^ cells/well. Posterior to cultivation for 300 min under 37 °C in an incubating device, the plates were studied via a microscopic device (Nikon, Japan). We obtained ≥5 fields for every matrix and the overall length of capillary tubes and quantity of branching points per field were calculated via a researcher blinded to our assay by virtue of NIH Image J 1.45 program (Bethesda, America).

### The release kinetics of Ginsenoside Rb1 from HAp

According to our previous study, the levels of drugs utilized in the bony defect model at the multiple of 100 folds of that in vitro could acquire the best osteogenesis effect.^2^ Ginsenoside Rb1 at the concentrations of 2000 μmol·L^−1^ were frozen in −80 °C overnight, and distillation was executed to realize the evaporation of the solvent DMSO (Sigma, America).

Then 1 mL SBF was supplemented to every compound and cultivated under 37 °C, subsequently, the supernate was harvested and preserved under 4 °C. At every chosen temporal point (1, 3, 6, 12, 24 h, 4, and 7 days). Afterward, the sample was subjected to resuspension in new SBF and cultivated till the following temporal point. The releasing of Ginsenoside Rb1 was subjected to quantification via the HPLC instrument (Shimadzu 2010C, America), and the data are described via the accumulative releasing as a function of the releasing time:

Accumulative quantity of release (%) = 100 × Mt/M∞ in which Mt denotes the quantity of Ginsenoside Rb1 generated from a specimen at temporal point t. The sum of Ginsenoside Rb1 in a specimen was computed and considered M∞ herein. We examined 3 specimens for every group and the outcomes were presented as mean values.

### Preparation of composite silk fibroin hydrogel gel with Ginsenoside Rb1 loaded HAp

Silk fibroin hydrogel gel was prepared as follow: The silkworm cocoons, purchased from Sigma-Aldrich (St. Louis, America), were cut and boiled for 30 min. The silk was washed thoroughly with deionized water. Posterior to degumming, the silk was subjected to dehydration in a drying device under 60 °C for 6 h and afterward subjected to dissolution in a CaCl_2_/EtOH/H_2_O solvent system under 100 °C for 20 min. Posterior to centrifuging, the supernate was move to the dialytic cassette and subjected to dialysis in deionization water for 3 d. The obtained aqueous silk liquor was afterward subjected to lyophilization under −80 °C to acquire the purified regenerated B. mori SF. Then the SF was dissoluted by deionized water, and the silk fibroin hydrogel gel in this study was 6%w/v.^15^

The micro-nano HAp particulates were prepared via the hydrotherm transform of the α-tricalcium phosphate (α-TCP, [α-Ca_3_(PO_4_)_2_]) particulates in CaCl_2_ water solution as per our research in the past. 0.1 g of the acquired micro-nano HAp particulates was soaked in 75 μL Ginsenoside Rb1 at 2000 μmol·L^−1^ nightlong, before lyophilization to realize the evaporation of the solvent DMSO (Sigma, America). Then the silk, and sodium alginate (SA) were produced as 6% (w/v), and 2% (w/v) stock solutions, respectively. Then, 100 μl silk liquor and 100 μL SA liquor were mixed to acquire a silk/SA mix. Moreover, 100 mg HAp particles were supplemented to the silk/SA mix and agitated for a uniformed distribution. The mixture was allocated into a cylinderic Teflon mold of 5 mm diameter.

For the animal experiment, 20 μl BMSCs were plated at 1 × 10^5^ cells per cm^3^ on the composite gelation. Then the mixture was preserved under 37 °C till gelatinization.

### Critical-size cranial defect study in vivo

A 5 mm diameter of full-thickness rat cranial bone defect is a commonly used model for evaluating the in vivo bone formation ability of the designed complexes.^46^ Then, 12 female 12-week-old Sprague-Dawley rats were acquired from our hospital’s Animal Center (PRC) for a cranial defect repair experiment, which was approved by Animal Experiment Ethic Board of our hospital affiliated to SJTU (HKDL[2016]321). Thereafter, the animals were separated into 4 groups, including silk fibroin hydrogel gel containing no Ginsenoside Rb1-loaded HAp granules (group A, silk/HAp, *n* = 3), silk fibroin hydrogel gel containing Ginsenoside Rb1-loaded HAp granules (group B, silk/HAp/Rb1, *n* = 3), BMSCs loaded silk fibroin hydrogel gel containing no Ginsenoside Rb1-loaded HAp granules (group C, BMSCs/silk/HAp, *n* = 3), and BMSCs loaded silk fibroin hydrogel gel containing Ginsenoside Rb1-loaded HAp granules (group D, BMSCs/silk/HAp/Rb1, *n* = 3).

### Sequential fluorescence labeling

In terms of the 8-week observation, a multicolor sequence fluorescence labeling for newly formed bones and mineralisation was completed. In short, via intraperitoneal injection, the rats were exposed to 25 mg/kg tetracycline hydrochloride (TE, Sigma, America), 30 mg·kg^−1^ alizarin red (AL, Sigma, America), and 20 mg·kg^−1^ Ca (Sigma), at 2, 4, and 6 weeks posterior to the treatment, separately.

### Histology and histomorphometry analysis

The cranial bone specimens were subjected to dehydration in elevating levels of alcohol from 70% to 100%, and afterward subjected to polymethylmethacrylate (PMMA) embedment. We fabricated 3 longitudinal slices for every sample as depicted in our past researches.^17^ Initially, the specimens were studied for fluorescence labeling via CLSM (Leica TCS, Germany), and the fluorochrome dyeing for newly formed bones and the quantification of mineralisation was achieved. The data on yellow (TE), red (AL), and green (CA) denote the osteogenesis and mineralisation at 2, 4, and 6 weeks posterior to operation, separately. Eventually, the specimens were dyed in Van Gieson’s picro fuchsin for histology analysis. The region of new bone formation was subjected to quantification from the serial slice harvested from every specimen, via Image Pro Plus 6.0, and presented as a proportion (%) of the entire bone defect region, separately.

### Statistics

Herein, the outcomes of repeatedly performed assays were described as the average ± SD. The remarkable diversity between datasets (*P < 0.05) was studied via one-way ANOVA.

## References

[1] Liu Y (2013). Integration of a calcined bovine bone and BMSC-sheet 3D scaffold and the promotion of bone regeneration in large defects. Biomaterials.

[2] Wu Y (2015). Evaluation of osteogenesis and angiogenesis of icariin loaded on micro/nano hybrid structured hydroxyapatite granules as a local drug delivery system for femoral defect repair. J. Mater. Chem. B.

[3] Siddiqi MH (2013). Ginseng saponins and the treatment of osteoporosis: mini literature review. J. Ginseng Res..

[4] Park EK (2005). Inhibitory effect of ginsenoside Rb1 and compound K on NO and prostaglandin E2 biosyntheses of RAW264.7 cells induced by lipopolysaccharide. Biol. Pharm. Bull..

[5] Wang CZ (2006). Steamed American ginseng berry: ginsenoside analyses and anticancer activities. J. Agric Food Chem..

[6] Cheng Y, Shen LH, Zhang JT (2005). Anti-amnestic and anti-aging effects of ginsenoside Rg1 and Rb1 and its mechanism of action. Acta Pharm. Sin..

[7] Qi LW, Wang CZ, Yuan CS (2011). Ginsenosides from American ginseng: chemical and pharmacological diversity. Phytochemistry.

[8] Jia JM, Wang ZQ, Wu LJ, Wu YL (2008). [Advance of pharmacological study on ginsenoside Rb1]. Zhongguo Zhong Yao Za Zhi.

[9] Keum YS (2000). Antioxidant and anti-tumor promoting activities of the methanol extract of heat-processed ginseng. Cancer Lett..

[10] Mohanan P, Subramaniyam S, Mathiyalagan R, Yang DC (2018). Molecular signaling of ginsenosides Rb1, Rg1, and Rg3 and their mode of actions. J. Ginseng Res.

[11] Wang C (2004). Phenotypic expression of bone-related genes in osteoblasts grown on calcium phosphate ceramics with different phase compositions. Biomaterials.

[12] Han YJ, Loo SC, Lee J, Ma J (2007). Investigation of the bioactivity and biocompatibility of different glass interfaces with hydroxyapatite, fluorohydroxyapatite and 58S bioactive glass. Biofactors.

[13] Vepari C, Kaplan DL (2007). Silk as a Biomaterial. Prog. Polym. Sci..

[14] Zhang W (2011). The use of injectable sonication-induced silk hydrogel for VEGF(165) and BMP-2 delivery for elevation of the maxillary sinus floor. Biomaterials.

[15] Zheng A (2018). Biocompatible silk/calcium silicate/sodium alginate composite scaffolds for bone tissue engineering. Carbohydr. Polym..

[16] Biswas T, Mathur AK, Mathur A (2017). A literature update elucidating production of Panax ginsenosides with a special focus on strategies enriching the anti-neoplastic minor ginsenosides in ginseng preparations. Appl. Microbiol. Biotechnol..

[17] Xie JT (2006). Antioxidant effects of ginsenoside Re in cardiomyocytes. Eur. J. Pharm..

[18] Tan S (2014). Anti-inflammatory effect of ginsenoside Rb1 contributes to the recovery of gastrointestinal motility in the rat model of postoperative ileus. Biol. Pharm. Bull..

[19] Wang Y (2018). Ginsenoside Rb1 inhibit apoptosis in rat model of Alzheimer’s disease induced by Abeta1-40. Am. J. Transl. Res.

[20] Zhao J, Lu S, Yu H, Duan S, Zhao J (2018). Baicalin and ginsenoside Rb1 promote the proliferation and differentiation of neural stem cells in Alzheimer’s disease model rats. Brain Res.

[21] Lo YT, Tsai YH, Wu SJ, Chen JR, Chao JC (2011). Ginsenoside Rb1 inhibits cell activation and liver fibrosis in rat hepatic stellate cells. J. Med. Food.

[22] Ducy P, Zhang R, Geoffroy V, Ridall AL, Karsenty G (1997). Osf2/Cbfa1: a transcriptional activator of osteoblast differentiation. Cell.

[23] Yang SH, Sharrocks AD, Whitmarsh AJ (2003). Transcriptional regulation by the MAP kinase signaling cascades. Gene.

[24] Celil AB, Campbell PG (2005). BMP-2 and insulin-like growth factor-I mediate Osterix (Osx) expression in human mesenchymal stem cells via the MAPK and protein kinase D signaling pathways. J. Biol. Chem..

[25] Jaiswal RK (2000). Adult human mesenchymal stem cell differentiation to the osteogenic or adipogenic lineage is regulated by mitogen-activated protein kinase. J. Biol. Chem..

[26] Cantley LC (2002). The phosphoinositide 3-kinase pathway. Science.

[27] Martelli AM (2006). Phosphoinositide 3-kinase/Akt signaling pathway and its therapeutical implications for human acute myeloid leukemia. Leukemia.

[28] Roohani-Esfahani SI, Nouri-Khorasani S, Lu Z, Appleyard R, Zreiqat H (2010). The influence hydroxyapatite nanoparticle shape and size on the properties of biphasic calcium phosphate scaffolds coated with hydroxyapatite-PCL composites. Biomaterials.

[29] Zhang W (2020). An all-silk-derived functional nanosphere matrix for sequential biomolecule delivery and in situ osteochondral regeneration. Bioact. Mater..

[30] Wang X, Yucel T, Lu Q, Hu X, Kaplan DL (2010). Silk nanospheres and microspheres from silk/pva blend films for drug delivery. Biomaterials.

[31] Kundu B, Rajkhowa R, Kundu SC, Wang X (2013). Silk fibroin biomaterials for tissue regenerations. Adv. Drug Deliv. Rev..

[32] Rockwood DN (2011). Materials fabrication from Bombyx mori silk fibroin. Nat. Protoc..

[33] Crouzier T (2011). The performance of BMP-2 loaded TCP/HAP porous ceramics with a polyelectrolyte multilayer film coating. Biomaterials.

[34] Przekora A, Ginalska G (2015). Enhanced differentiation of osteoblastic cells on novel chitosan/beta-1,3-glucan/bioceramic scaffolds for bone tissue regeneration. Biomed. Mater..

[35] Crisan M (2008). A perivascular origin for mesenchymal stem cells in multiple human organs. Cell Stem Cell.

[36] Schott, N. G., Friend, N. E. & Stegemann, J. P. Coupling osteogenesis and vasculogenesis in engineered orthopedic tissues. *Tissue Eng. Part B Rev.* **27**, 199–214 (2021). 10.1089/ten.teb.2020.0132 PMC8349721 32854589

[37] Bulnheim U (2014). Endothelial cells stimulate osteogenic differentiation of mesenchymal stem cells on calcium phosphate scaffolds. J. Tissue Eng. Regen. Med..

[38] Shanbhag S, Pandis N, Mustafa K, Nyengaard JR, Stavropoulos A (2017). Cell cotransplantation strategies for vascularized craniofacial bone tissue engineering: a systematic review and meta-analysis of preclinical in vivo studies. Tissue Eng. Part B Rev..

[39] Bakshi R (2019). Application of hydroxycholesterols for alveolar cleft osteoplasty in a rodent model. Plast. Reconstr. Surg..

[40] Pou AM (2003). Update on new biomaterials and their use in reconstructive surgery. Curr. Opin. Otolaryngol. Head. Neck Surg..

[41] Kao RT, Conte G, Nishimine D, Dault S (2005). Tissue engineering for periodontal regeneration. J. Calif. Dent. Assoc..

[42] Guerrero J (2013). Cell interactions between human progenitor-derived endothelial cells and human mesenchymal stem cells in a three-dimensional macroporous polysaccharide-based scaffold promote osteogenesis. Acta Biomater..

[43] Sun Z, Cai S, Zabkiewicz C, Liu C, Ye L (2020). Bone morphogenetic proteins mediate crosstalk between cancer cells and the tumour microenvironment at primary tumours and metastases (Review). Int J. Oncol..

[44] Wu QJ (2014). Antibacterial property, angiogenic and osteogenic activity of Cu-incorporated TiO_2_ coating. J. Mater. Chem. B.

[45] Wu Y (2013). Effect of mechanical stretch on the proliferation and differentiation of BMSCs from ovariectomized rats. Mol. Cell Biochem.

[46] Wu, Q. et al. Study of Sr–Ca–Si-based scaffolds for bone regeneration in osteoporotic models. *Int. J. Oral Sci.* **12**, 25 (2020). 10.1038/s41368-020-00094-1 PMC7505977 32958751

